# Network Analyses of Differentially Expressed Genes in Osteoarthritis to Identify Hub Genes

**DOI:** 10.1155/2019/8340573

**Published:** 2019-04-16

**Authors:** Zhaoyan Li, Lei Zhong, Zhenwu Du, Gaoyang Chen, Jing Shang, Qiwei Yang, Qingyu Wang, Yang Song, Guizhen Zhang

**Affiliations:** ^1^Department of Orthopedics of the Second Hospital of Jilin University, Ziqiang Street 218, Changchun, Jilin 130041, China; ^2^Research Centre of the Second Hospital of Jilin University, Ziqiang Street 218, Changchun, Jilin 130041, China; ^3^The Engineering Research Centre of Molecular Diagnosis and Cell Treatment for Metabolic Bone Diseases of Jilin Province, Ziqiang Street 218, Changchun, Jilin 130041, China

## Abstract

**Background:**

Osteoarthritis (OA) is the most common degenerative disease in orthopedics. However, the cause and underlying molecular mechanisms are not clear. This study aims to identify the hub genes and pathways involved in the occurrence of osteoarthritis.

**Methods:**

The raw data of GSE89408 were downloaded from the Gene Expression Omnibus (GEO) database, and the differentially expressed genes (DEGs) were identified by R software. The DAVID database was used for pathway and gene ontology analysis, and p<0.05 and gene count >2 were set as the cut-off point. Moreover, protein-protein interaction (PPI) network construction was applied for exploring the hub genes in osteoarthritis. The expression levels of the top ten hub genes in knee osteoarthritis synovial membranes and controls were detected by quantitative real-time PCR system.

**Results:**

A total of 229 DEGs were identified in osteoarthritis synovial membranes compared with normal synovial membranes, including 145 upregulated and 84 downregulated differentially expressed genes. The KEGG pathway analysis results showed that up-DEGs were enriched in proteoglycans in cytokine-cytokine receptor interaction, chemokine signaling pathway, rheumatoid arthritis, and TNF signaling pathway, whereas down-DEGs were enriched in the PPAR signaling pathway and AMPK signaling pathway. The qRT-PCR results showed that the expression levels of ADIPOQ, IL6, and CXCR1 in the synovium of osteoarthritis were significantly increased (p <0.05).

## 1. Introduction

Osteoarthritis is the most common degenerative disease worldwide; the global incidence of knee osteoarthritis is estimated at 3.8 percent in 2010 [1]. Osteoarthritis is the leading cause of disability in the US, with 43.5% of the arthritis patients having activity limitations [2]. Pain and stiffness, especially post-exercise pain and stiffness, are the main symptoms and have a considerable impact on the ability of daily life activities[1]. These symptoms severely reduce the quality of life and create a heavy socioeconomic burden.

Recent research has increasingly recognized the important role of the synovial membrane in the progression of osteoarthritis [3]. Synovitis in osteoarthritis is associated with activity disorder, joint severe pain, and cartilage loss in certain patient populations [4]. Osteoarthritis is a polygenic disorder. In recent years, genetic analysis data have revealed that OA-related genes are often associated with the development of synovial joints [5]. But until now, the hub gene and pathway of osteoarthritis are still not clear. Therefore, it is critical to elucidate the pathogenesis and progression of osteoarthritis further.

In this work, we have downloaded the microarray dataset GSE89408 from the GEO database, which contained 22 knee osteoarthritis synovial membranes and 28 matched normal synovial membranes. DEGs in osteoarthritis and normal synovial membranes were identified using the limma package in RStudio software, and DAVID database was used for pathway and gene ontology analysis of DEGs. Then, the PPI network was constructed to show the connection of DEGs and screening of hub genes. Our study aims to explore the hub genes and pathways of osteoarthritis, which may help to better understand the molecular mechanism of osteoarthritis.

## 2. Materials and Methods

### 2.1. Microarray Data Information

The GEO database is a public genomics data repository which provides high-throughput gene expression data [6]. The raw data of GSE89408 (GPL11154 platform, Illumina HiSeq 2000) was downloaded from the GEO database which includes 22 synovial membranes of knee osteoarthritis and 28 normal synovial membranes of controls [7].

### 2.2. Data Preprocessing and DEG Screening

The limma package in R was used to identify the DEGs between osteoarthritis synovial membranes and normal synovial membranes [8]. Background adjustment, normalization, and summarization were all included in the process of preprocessing. P values were adjusted using the Hochberg and Benjamini test, and p<0.05 and gene count >2 were set as the cut-off point.

### 2.3. Gene Ontology and Pathway Analysis

The DAVID Gene Functional Classification Tool can classify large genes into biological modules [9]. Gene Ontology provides the logical structure of the biological functions and their relationships to one another genes. GO analysis includes categories of molecular function (MF), cellular component (CC), and biological processes (BP). Pathway analysis is the process of classifying large genes by the KEGG database. In our study, candidate DEGs functions and pathway enrichment were analyzed using the DAVID database. P<0.05 and gene count >2 were set as the cut-off point.

### 2.4. PPI Network Integration

The STRING database is an online resource whose main function is to construct functional protein association networks [10]. The interaction scores >0.40 (medium confidence) were defined as significant. The PPI was constructed by Cytoscape software, and the interaction relationship of DEGs was analyzed by a plug-in of Cytoscape software. CytoHubba, as a plug-in Cytoscape software, provides an effective method for identifying hub genes in PPI networks through degree method [11].

### 2.5. qRT-PCR Validation and Statistical Analysis

Hub genes were verified by the qRT-PCR system. Total RNA was extracted from the control and knee osteoarthritis synovial membranes using TRIzol reagent (TaKaRa, Japan) and then reverse-transcribed to cDNA. Primers were designed by Primer 5.0 software (PREMIER Biosoft, Palo Alto, CA, USA), and a QuantStudio™ 7 Flex real-time PCR system (Applied Biosystems, Carlsbad, CA, USA) was used. Primers for mRNA are shown in Table 1. All samples were normalized to GAPDH. And the relative expression levels of each gene were calculated using 2−ΔΔCt methods. SPSS software (version 22.0 SPSS Inc.) was utilized to analyze statistical data, and P values < 0.05 were considered as statistically significant.

### 2.6. Patients and Controls

Our research was approved by the Ethics Committee of the Second Hospital of Jilin University, Jilin, China. 10 knee osteoarthritis patients and 10 meniscal tear patients without obvious synovitis were enrolled, and all gave informed consent. Synovial samples of knee osteoarthritis were obtained from patients undergoing total knee arthroplasty in the Second Hospital of Jilin University, Jilin, China. And normal synovial membranes were obtained from arthroscopic surgery cases upon meniscus injury in the Second Hospital of Jilin University, Jilin, China.

## 3. Results

### 3.1. Differentially Expressed Genes

**Figure 1:**
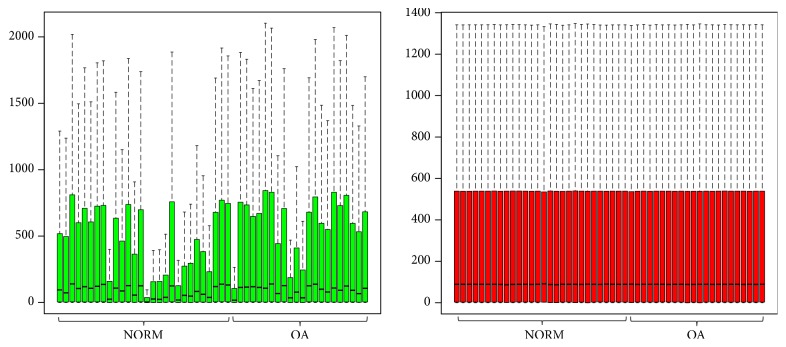
Gene expression before and after normalization: The green box plots represent the data before normalization, and the red box plots represent the normalized data.

The GSE89408 dataset was standardized, and the results are shown in Figure 1. A total of 22 knee osteoarthritis synovial membranes and 28 matched normal samples were analyzed, using |log FC| > 2 and p < 0.05 as the cut-off criteria, and we ultimately obtained 229 DEGs in osteoarthritis synovial membranes compared with normal synovial membranes, including 145 upregulated and 84 downregulated DEGs.

### 3.2. Enrichment Analysis of DEGs

**Figure 2:**
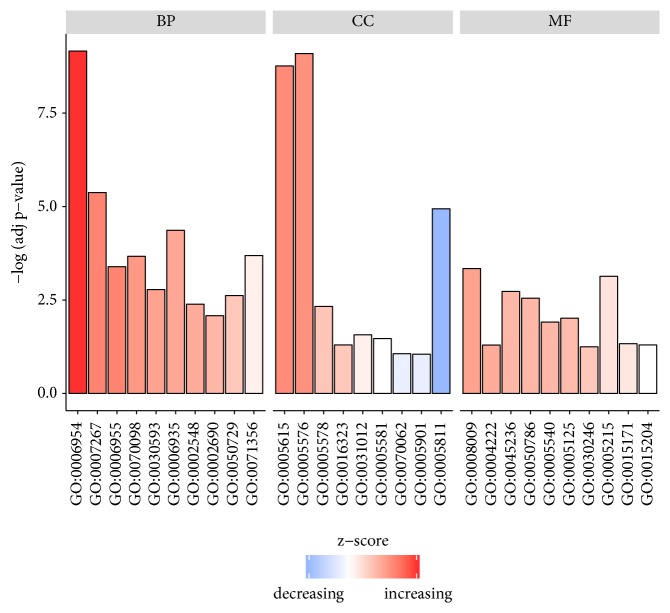
The top ten GO terms in each group: The top ten GO terms in each group were ranked by p value. The gradual color represents the z-score.

**Figure 3:**
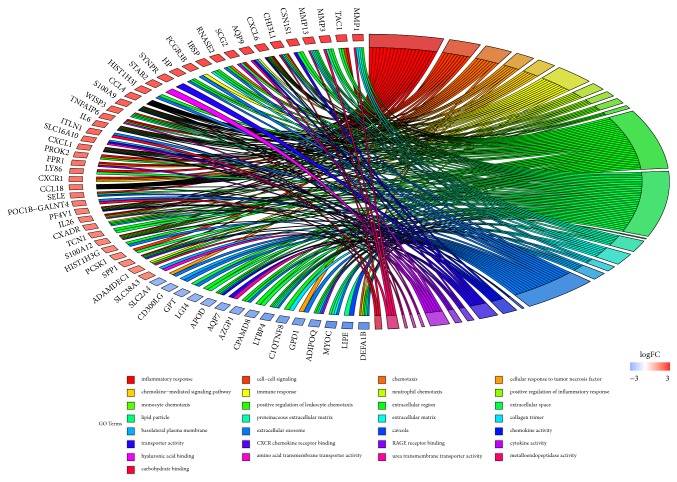
GO enrichment analysis of DEGs: The gradual color represents the log⁡FC. The genes were ordered according to their log⁡FC values setting gene.

**Figure 4:**
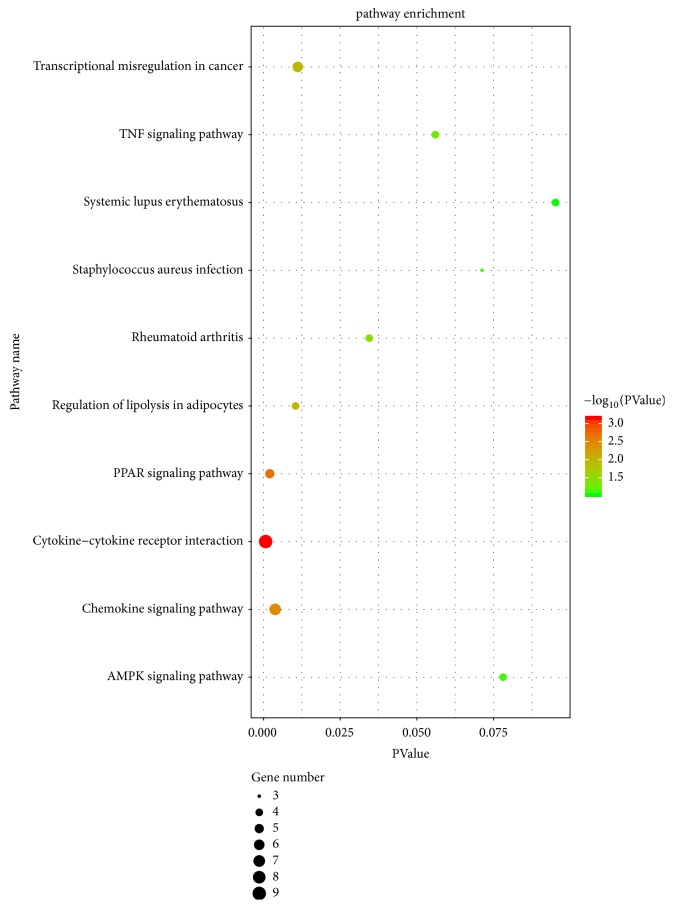
KEGG enrichment analysis of the pathways: The gradual color represents the P value, the size of the black spots represents the gene number.

To acquire the functions of DEGs, GO function enrichment was analyzed by DAVID database. The top five BP terms, CC terms, and MF terms are shown in Table 2. In the BP group, the up-DEGs are mainly enriched in the inflammatory response, cell-cell signaling, chemokine-mediated signaling pathway, and immune response, and the down-DEGs are mainly enriched in lipid metabolic process and brown fat cell differentiation. In the CC group, the up-DEGs are mainly enriched in the extracellular region and extracellular space, and the down-DEGs are mainly enriched in lipid particle and mitochondrion. Moreover, in the MF group, the up-DEGs are mainly enriched in CXCR chemokine receptor binding, RAGE receptor binding, cytokine activity, and chemokine activity, and the down-DEGs are mainly enriched in drug binding and protein homodimerization activity. The results of GO enrichment analysis are shown in Figures 2 and 3. The pathway analysis results showed that up-DEGs were enriched in proteoglycans in cytokine-cytokine receptor interaction, chemokine signaling pathway, rheumatoid arthritis, and TNF signaling pathway, whereas down-DEGs were enriched in the PPAR signaling pathway and AMPK signaling pathway. The results are shown in Figure 4 and Table 3.

### 3.3. PPI Network Analysis and Hub Genes Screening

**Figure 5:**
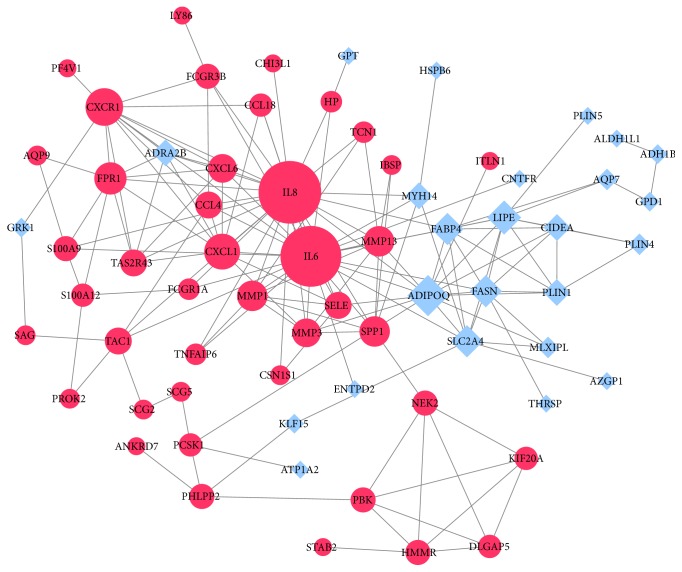
PPI network of DEGs: Red circles represent upregulated DEGs, and blue squares represent downregulated DEGs. The size of the nodes represents the degree value.

**Figure 6:**
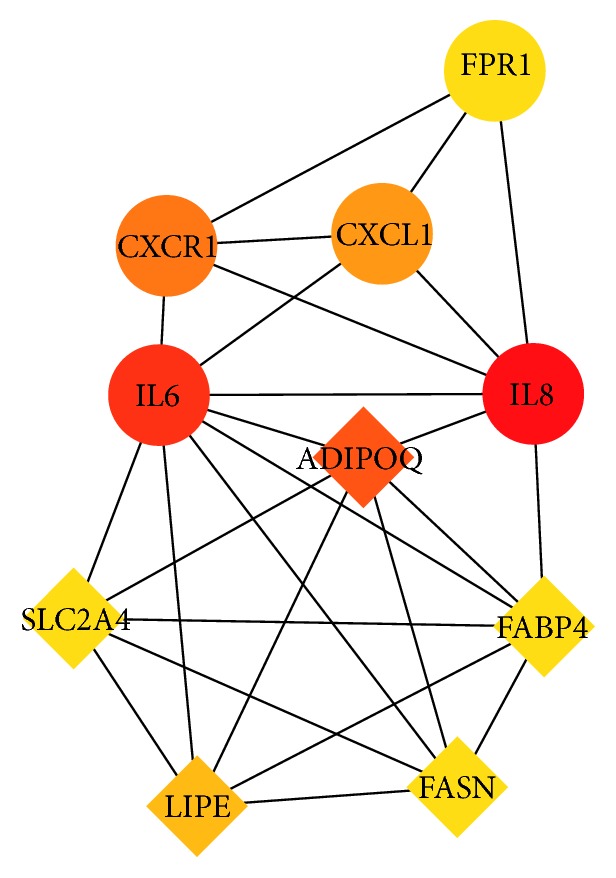
PPI network of hub genes: The gradual color represents the degree value. The circles represent upregulated genes, and squares represent downregulated genes.

Using the STRING database and Cytoscape software, we get a total of 67 nodes including 42 upregulated and 25 downregulated DEGs (Figure 5). Through plug-in CytoHubba in Cytoscape software, we evaluated the degree and betweenness centrality in the PPI network and screening the hub genes. The 10 hub genes showing significant interaction were IL8, ADIPOQ, CXCR1, CXCL1, LIPE, FPR1, FABP4, SLC2A4, FASN, and IL6 (Figure 6).

### 3.4. Validation of Hub Genes

**Figure 7:**
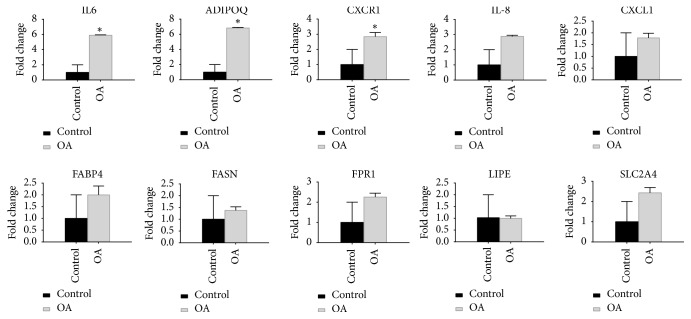
Validation of the top 10 hub genes by qRT-PCR between the OA group and the control group: All samples were normalized to GAPDH. And the relative expression levels of each gene were calculated using 2−ΔΔCt methods. *∗* represents P <0.05.

To verify the results of microarray, the expression levels of the top ten hub genes in knee osteoarthritis synovial membranes and controls were detected by the qRT-PCR system. The statistical results showed that the expression levels of ADIPOQ, IL6, and CXCR1 in the synovium of osteoarthritis were significantly increased (p <0.05) (Figure 7). All validations are consistent with the analytical results in this study.

## 4. Discussion

Osteoarthritis is the most common degenerative disease worldwide that affects small and large joints. Osteoarthritis often affects the whole joint tissue, including synovium, subchondral bone, and cartilage [12]. Synovitis is a common feature of osteoarthritis, even in early diseases. Synovial proliferation and tissue hypertrophy are significant in advanced osteoarthritis. There is increasing evidence that synovitis plays a key role in the pathogenesis and progression of osteoarthritis. Further understanding of the molecular and cellular variability of osteoarthritis-related synovitis can provide insight into the etiology of arthritis [4]. GWAS studies help to determine the important role of genetic factors in the risk of osteoarthritis. Understanding the key genes that influence the onset and progression of osteoarthritis will be necessary for the development of early treatment of the disease.

In our study, the analysis of gene expression profiling revealed the hub genes and pathways associated with osteoarthritis and enabled the identification of targets for therapeutic strategy. Bioinformatics methods are applied to analyze the raw data, and we identify 229 DEGs in osteoarthritis synovial membranes compared with normal synovial membranes, including 145 upregulated DEGs and 84 downregulated DEGs. Next, all DEGs were classified into three groups by GO terms using the online database and further clustered based on gene functions and signaling pathways, respectively. The PPI network of DEGs was constructed by STRING database, and the top ten hub genes were obtained: IL8, ADIPOQ, CXCR1, CXCL1, LIPE, FPR1, FABP4, SLC2A4, FASN, and IL6. The statistical result of the verification experiment showed that the expression level of ADIPOQ, IL6, and CXCR1 was significantly increased in knee osteoarthritis synovial membranes (p < 0.05). Studying these hub genes may contribute to the early diagnosis and treatment of osteoarthritis.

IL-6 plays a crucial role in chronic inflammation and the expression levels of IL-6 increase in human inflammatory diseases. In acute and chronic inflammation, IL-6 secreted into the serum and induced a transcriptional inflammatory response through interleukin 6 receptor alpha [13]. IL-6 expression was increased in osteoarthritis synovial membranes and synovial fluid, histone hyperacetylation, and DNA hypomethylation in the promoter of IL-6 gene were observed in osteoarthritis synovial fibroblasts compared with healthy synovial fibroblasts. Overexpression of IL-6 in synovial fibroblasts of osteoarthritis was suppressed through decrease histone acetylation and overexpression DNA methylation [14]. In our study, IL6 was significantly increased in knee osteoarthritis synovial membranes. The KEGG pathway enrichment showed that IL-6 was enriched in rheumatoid arthritis, cytokine-cytokine receptor interaction, and TNF signaling pathway [15, 16]. Previous studies showed that these pathways are involved in osteoarthritis synovial hyperplasia. Inflammatory cytokines, including IL-1*β*, TNF*α*, and IL-6, play a key role in osteoarthritis, and IL-6 is considered to be the key cytokine; its effect is mainly based on promoting the formation of osteoclasts and bone resorption while synergism with IL-1*β* and TNF*α* [15]. In our previous research, the expression levels of IL6 and IL-1*β* were significantly increased in osteoarthritis synovial membranes [17]. Chemokine is a small secretory molecule, which mainly plays the role of chemotactic immune cells. The chemokine receptors CXCR1 and CXCR2 play a role in neutrophil-dependent injury and mediating neutrophil recruitment in inflammatory disease [18]. CXCR1/CXCR2 play a significant role in regulating tissue inflammation in a mouse model of osteoarthritis [19]. Cytokine networks in osteoarthritis may be the future research direction in therapeutic strategies. Different from microarray analysis results, ADIPOQ was significantly increased in knee osteoarthritis synovial membranes in our study. Adiponectin is a specific protein expressed in adipose tissue exclusively. The previous study found that SNP rs182052 is significantly associated with susceptibility to knee osteoarthritis in the Chinese population [20], and the interaction between rs1501299 (ADIPOQ) and rs662 (PON1) gene polymorphisms may play an important role in the development of osteoarthritis [21]. And the expression level of adiponectin was significantly higher in osteoarthritis patients than in controls [22]. In a word, ADIPOQ gene mutation may be associated with an increased risk of knee osteoarthritis.

In summary, using integrated bioinformatical analysis and qRT-PCR validation, we have identified the hub genes and related pathways, and these findings have the potential to be used as biomarkers and targets for osteoarthritis early diagnosis and treatment. There are still many limitations in our research: small sample size and lack of further experiments. To confirm our analysis results, more experimental studies with a larger sample are needed to confirm our study.

## Figures and Tables

**Table 1:** The primers of top 10 hub genes.

Gene	Forward primer	Reverse primer
GAPDH	CGGACCAATACGACCAAATCCG	AGCCACATCGCTCAGACACC
IL8	ACTGAGAGTGATTGAGAGTGGAC	AACCCTCTGCACCCAGTTTTC
Il6	TCAATATTAGAGTCTCAACCCCCA	GAAGGCGCTTGTGGAGAAGG
ADIPOQ	GCTGGGAGCTGTTCTACTG	TACTCCGGTTTCACCGATGTC
CXCR1	CTGACCCAGAAGCGTCACTTG	CCAGGACCTCATAGCAAACTG
CXCL1	AACCGAAGTCATAGCCACAC	GTTGGATTTGTCACTGTTCAGC
LIPE	TCAGTGTCTAGGTCAGACTGG	AGGCTTCTGTTGGGTATTGGA
SLC2A4	TGGGCGGCATGATTTCCTC	GCCAGGACATTGTTGACCAG
FASN	AAGGACCTGTCTAGGTTTGATGC	TGGCTTCATAGGTGACTTCCA
FABP4	ACTGGGCCAGGAATTTGACG	CTCGTGGAAGTGACGCCTT
FPR1	TGGGAGGACATTGGCCTTTC	GGATGCAGGACGCAAACAC

**Table 2:** The significantly enriched analysis of DEGs in osteoarthritis.

Expression	Category	Term	Description	Gene Count	P Value
UP-DEGs	BP	GO:0006954	inflammatory response	19	1.21E-13
	BP	GO:0007267	cell-cell signaling	11	3.03E-07
	BP	GO:0070098	chemokine-mediated signaling pathway	6	2.07E-05
	BP	GO:0006935	chemotaxis	7	2.34E-05
	BP	GO:0006955	immune response	11	2.71E-05
	CC	GO:0005615	extracellular space	25	1.66E-09
	CC	GO:0005576	extracellular region	27	2.28E-09
	CC	GO:0005578	proteinaceous extracellular matrix	6	0.006499621
	CC	GO:0016323	basolateral plasma membrane	4	0.044790466
	CC	GO:0031988	membrane-bounded vesicle	2	0.05975494
	MF	GO:0008009	chemokine activity	5	6.54E-05
	MF	GO:0045236	CXCR chemokine receptor binding	3	6.87E-04
	MF	GO:0050786	RAGE receptor binding	3	0.001043791
	MF	GO:0005540	hyaluronic acid binding	3	0.004638666
	MF	GO:0005125	cytokine activity	5	0.007811921

DOWN-DEGs	BP	GO:0006629	lipid metabolic process	5	0.001123386
	BP	GO:0050873	brown fat cell differentiation	3	0.003912643
	BP	GO:0010890	positive regulation of sequestering of triglyceride	2	0.020252031
	BP	GO:0010642	negative regulation of platelet-derived	2	0.020252031
			growth factor receptor signaling pathway		
	BP	GO:0042593	glucose homeostasis	3	0.035075126
	CC	GO:0005811	lipid particle	7	6.93E-08
	CC	GO:0005739	mitochondrion	11	0.009807547
	CC	GO:0070062	extracellular exosome	16	0.031369825
	CC	GO:0005576	extracellular region	11	0.032718894
	CC	GO:0005615	extracellular space	9	0.067564494
	MF	GO:0042803	protein homodimerization activity	7	0.016759119
	MF	GO:0008144	drug binding	3	0.01974095

**Table 3:** Signaling pathway enrichment analysis of DEGs function in osteoarthritis.

Expression	Term	Description	Gene Count	P Value
DOWN-DEGs	hsa04923	Regulation of lipolysis in adipocytes	4	7.05E-04
	hsa03320	PPAR signaling pathway	4	0.001191375
	hsa04152	AMPK signaling pathway	4	0.006702552
	hsa04910	Insulin signaling pathway	3	0.07107271

UP-DEGs	hsa04060	Cytokine-cytokine receptor interaction	8	1.22E-04
	hsa04062	Chemokine signaling pathway	6	0.001756326
	hsa05202	Transcriptional misregulation in cancer	5	0.007918451
	hsa05323	Rheumatoid arthritis	4	0.008366703
	hsa04668	TNF signaling pathway	4	0.014229192
	hsa05322	Systemic lupus erythematosus	4	0.02575911
	hsa05150	Staphylococcus aureus infection	3	0.027341561

## Data Availability

The microarray data used to support the findings of this study have been deposited in the GEO database (dataset ID:GSE89408), which have been cited.
